# Peanut Coffee: Enhancement of Nutritional, Physicochemical, and Sensory Characteristics in Coffee Brewed with Conventional and High-Oleic Peanut Extracts

**DOI:** 10.3390/foods9111664

**Published:** 2020-11-14

**Authors:** Jookyeong Lee, Seong Jun Hong, Jin-Ju Cho, Chang Guk Boo, Da-Som Kim, Eui-Cheol Shin

**Affiliations:** Department of Food Science, Gyeongnam National University of Science and Technology, Jinju 52725, Korea; tracylee0911@gmail.com (J.L.); 01028287383a@gmail.com (S.J.H.); aacho7@hanmail.net (J.-J.C.); dbs7987@naver.com (C.G.B.); kim94dasom@naver.com (D.-S.K.)

**Keywords:** coffee, peanuts, nutrients, electronic tongue, volatile compounds, sniffing test

## Abstract

This study investigated nutritional, physicochemical, and sensory characteristics of coffee brewed with conventional and high-oleic peanut extracts. Compared to normal coffee, peanut coffee exhibited more diverse amino acids compositions. In constituent amino acids composition, peanut coffee exhibited increased proportions of glutamic and aspartic acids but decreased phenylalanine. Peanut coffee had higher thiamin, niacin, and sugar contents, improved antioxidant capacity, and lower caffeine contents. In electronic tongue analysis, peanut coffee showed an increased intensity of sweetness and umami taste but decreased bitterness, corresponding to the result of amino acids compositions and caffeine and sugar contents. In volatile compounds analysis using gas chromatography-mass spectrometry (GC/MS) with the sniffing test, peanut coffee had high concentrations of 2-ethyl-3,6-dimethylpyrazine, 2,2′-methylenebis-furan, and furfuryl propionate, which were perceived as peanut and roasted odors in the sniffing test. This study will provide informative data in extending the application of peanut to coffee and developing novel coffee, with added peanut, that is nutritionally beneficial.

## 1. Introduction

Coffee is a favorite beverage that is widely consumed in modern society, and the demand for high quality specialty coffee has been increased by millions of people. In recent years, consumers have raised their interest to gourmet coffee such as *kopi Luwak*, while the market for instant coffee shows a gradual downtrend [1,2].

Peanut is a legume rich in lipids (50%), proteins (25%), carbohydrates (13%), and vitamins and minerals. Physiologically, peanut is effective in decreasing body weight [3], blood pressure, and blood cholesterol levels, and exhibits anti-cancerous and anti-oxidative effects, and therefore has been widely utilized in healthy foods and processed foods such as cooking oil and peanut butter [4]. For the sake of breeding improvements, high-oleic peanut has been developed since the 1980s. In fatty acid compositions, conventional peanut consists of approximately 55% oleic acid and 25% linoleic acid whereas high-oleic peanut contains 80% and 5%, respectively [3].

Volatile compounds are of paramount importance in developing coffee flavor. Coffee includes over 1000 volatile compounds, among which 300 homo- and hetero- cyclic compounds are responsible for the unique coffee flavor. Volatile compound profiles vary upon roasting intensity, variety, and origin of coffee beans [2,5]. Commonly accepted coffee flavor is generated through the roasting of coffee beans. Volatile compounds are developed via Maillard reaction, Strecker degradation, lipid and sugar decomposition, free amino acids loss, and decrease in free sugar, sucrose, chlorogenic acid, and trigonelline [6]. Low molecular weight volatile compounds are produced depending on the degree of roasting, resulting in light and positive flavor [2]. Volatile compounds in coffee are comprised of about 210 bases, 120 furans, 100 carbonyls, 90 sulfur compounds, 70 hydrocarbons, 50 phenols, 30 esters, 20 alcohols, and some acetals, amides, ethers, nitriles, and pyrans [2]. Most of the current knowledge on coffee volatiles was obtained through extraction using the solid-phase microextraction (SPME) and headspace method and analysis and identification using gas chromatography (GC) and gas chromatography/mass spectrometry (GC-MS) [7].

Taste is another critical sensory characteristic in coffee. Basic tastes consist of saltiness, sweetness, bitterness, sourness, and umami taste, with fat taste, which is known as the 6^th^ taste, recently identified [8]. Electronic tongues have been increasingly utilized for the purpose of quality control in the food industry. Electronic tongues possess sensors that imitate human taste perception, thereby being considered as having potential as a replacement for coffee taste analysis in a time- and cost-effective manner [9]. Electronic tongue analysis is a simple non-destructive pattern analysis method without pretreatment and provides relatively objective and reproducible results [10].

Many studies have investigated different aspects of coffee: antioxidant capacities of coffee bean and coffee residues [11], enhancing Robusta coffee aroma by modifying flavor precursors in the green coffee bean [12], chlorogenic acid and caffeine contents in various commercial brewed coffee [13], physicochemical properties and volatile compounds by roasting conditions of coffee bean [6], benzopyrene contents by roasting [14], and fatty acid compositions contained in instant coffee [15]. 

Whereas many studies investigated volatile compounds, antioxidant capacities, physicochemical properties, and hazard substances with variations in coffee bean types and extraction methods, only few studies demonstrated changes in nutritional and physicochemical properties and tastes/flavors by adding other food sources (e.g., peanut) to coffee. As aforementioned, peanut is a nutritionally and physiologically exceptional food source, but its utilization is somewhat limited to mostly snacks. Therefore, in this study, coffee was prepared with peanut extracts using conventional peanut and high-oleic peanut, and comparisons were made in terms of amino acids compositions, water-soluble vitamins contents, physicochemical characteristics, tastes by electronic tongue, and volatile compounds with the sniffing test. This study will be an exemplary attempt at extending the utilization of peanut to coffee and developing novel coffee with peanut added.

## 2. Materials and Methods

### 2.1. Materials and Sample Preparation

Coffee bean used in this study (Columbia) was produced by Bean Market (Gwangju, Republic of Korea) and purchased in a local grocery store in Jinju, Republic of Korea (Item number: 2012036853615). Conventional peanut variety (*Daekwang*) and high-oleic peanut variety (*K-ol*) were provided from National Institute of Crop Science (Milyang, Korea).

Peanut was roasted at 200 °C for 17 min using a roaster (CBR-101A, Genecafe Co, Ansan, Republic of Korea). Coffee bean was ground using a coffee grinder (KG79, Delonghi Co, Treviso, Italy) into medium size (600 μm). Peanut extract (5 g/100 mL) was prepared with water using a coffee maker (CM151GKR, Tefal Co, Rumilly, Haute-savoie, France). Coffee (3.75 g/100 mL) was extracted with the prepared peanut extract using the same coffee maker for 20 min. Three kinds of samples (normal coffee, conventional peanut coffee, and high-oleic peanut coffee) were prepared upon extraction of solvent (water, conventional peanut extract by a coffee maker, and high-oleic peanut extract by a coffee maker, respectively).

### 2.2. Nutritional Characteristics

Constituent amino acids were analyzed via a acid hydrolysis method using hydrochloric acid (HCl). One hundred milligrams of sample was added to 3 mL of 6 N HCl in a test tube. Acid hydrolysis was carried out at the following conditions: 110 °C for 24 h while stirring (150 rpm). The mixture (10 μL) was then diluted with sodium dilution buffer (990 μL) and filtered using a 0.2 μm membrane filter. An amino acid analyzer (L-8900, Hitachi High Tech, Tokyo, Japan) was utilized to analyze the constituent amino acid profiles [16]. Free amino acids analysis was conducted by adding 500 mg of sample to 10 mL of ethanol and stirring for 10 min. The solution was subjected to centrifugation at 3000 rpm for 20 min. The supernatant was then flushed with a stream of nitrogen and dissolved in a 12.5 mL lithium loading buffer. Then, 20 mg of sulfosalicylic acid was added to the solution, which was incubated at 4 °C for 1 h, followed by centrifugation at 3000 rpm for 20 min. The solution was filtered using a 0.2 μm membrane and the composition was examined by the same analyzer used for the constituent amino acid profile (L-8900, Hitachi High Tech) [16].

Agilent 1100 infinity HPLC with a diode array detector (Agilent Co., Santa Clara, CA, USA) was utilized to determine the contents of water-soluble vitamins with a column, YMC-Pack octadecylsilyl silica gel (ODS) AM (250 × 4.6 mm, 5 μm) (YMC-Korea Co., Seongam, Korea) at 40 °C. The detector was set at 270 nm. The mobile phase solvents were prepared as follows: solvent A was prepared with 7.5 mL acetic acid, 0.2 mL triethylamine, and 5 mM sodium 1-hexanesulfonate, and solvent B was methanol. A linear gradient elution was taken for this analysis. The mobile phase was flowed at 0.8 mL/min and then subsequently flowed at the following conditions: 0 min: 100% solvent A, 8 min: 100% solvent A, 20 min: 75% solvent A and 25% solvent B, 30 min: 55% solvent A, 31 min: 100% solvent A, 45 min: 100% solvent A. The same instrument with an identical column to that used in water-soluble vitamins analysis was used to analyze riboflavin.. The wavelengths of the detector were set to 445 nm for excitation and 530 nm for emission. The mobile phase was prepared with 10 mM NaH_2_PO_4_ at pH 5.5 and methanol (75:25, *v*/*v*), and the analysis was flowed at a rate of 0.8 mL/min in an isocratic elution condition [16].

Glucose and sucrose contents were determined using the Agilent 1260 high-performance liquid chromatography (HPLC) system (Agilent Co., Santa Clara, CA, USA) with a ZORBAX Carbohydrate (4.6 mm × 250 mm, 5 μm) column. Tertiary distilled water was used as mobile phase at 0.5 mL/min. The column was set at 50 °C and an refractive index (RI) (Agilent Co., Santa Clara, CA, USA) detector was used. 

Caffeine contained in coffee was determined using high performance liquid chromatography (HPLC) (Agilent Co., Santa Clara, CA, USA). Samples required no pretreatment for HPLC analysis. The column used was a ZORBAX Eclipse XDB-C18 (150 mm × 4.6 mm, 5 μm, YMC-Korea Co., Seongam, Korea), which was used at 40 °C. The detector was set at 270 nm. Sixty percent acetonitrile was used as mobile phase at 0.5 mL/min.

### 2.3. Physicochemical Characteristics 

Antioxidant capacity was measured by 2,2 diphenyl-1-picrylhydrazyl (DPPH) radical scavenging activity and total phenolic contents. DPPH radical scavenging activity was measured by a method outlined by Blois [17]. The sample was subjected to a series of dilution with distilled water (0.1, 1, and 10 mg/mL). Next, 10 μL of diluted sample was taken and mixed with 1 mL of 0.1 mM DPPH (dissolved in 99% ethanol, Sigma-Aldrich Co., St. Louis, MO, USA), followed by incubation in the dark at 37 °C for 30 min. Absorbance was measured at 517 nm and radical scavenging activity was determined by the Equation (1).

DPPH radical scavenging activity (%) = (1 − Sample absorbance/blank absorbance) × 100(1)

Based on the radical scavenging activity (%), SC_50_ (the sample concentration where radical scavenging activity reaches 50%) was calculated.

The Folin–Ciocalteu’s method was employed to determine total phenolic content [18]. After diluting the sample to 1 mg/mL with distilled water, 40 μL of the diluted solution was taken to further mix with 200 μL of distilled water, and 200 μL of 2 N Folin–Ciocalteu’s reagent (Sigma-Aldrich Co., St. Louis, MO, USA) was added and mixed. A quantity of 600 μL of 30% Na_2_CO_3_ and 160 μL of distilled water were then added to the solution and incubated at room temperature for 2 h. Total phenolic content was determined at 750 nm. As a standard, gallic acid (0–500 μg/mL) was utilized. Total phenolic content was calculated based on the calibration curve.

For pH measurement, 5 mL of sample was added to 45 mL of distilled water and 10 mL of the mixture was used to measure pH using a pH meter (ST3100, Ohaus Co, Parsippany, NJ, USA).

### 2.4. Sensory Characteristics

Volatile compounds in coffee samples were collected by solid phase microextraction (SPME) fiber coated with 100 μL polydimethylsiloxane (PDMS) (Supelco, Bellefonte, PA, USA). Pentadecane (10 μg in 1 mL of ethyl ether) was used as an internal standard. A total of 115 g of coffee sample was heated in a 60 °C heating block for 20 min and the volatile compounds were collected for 30 min in the SPME fiber. The collected compounds were introduced to desorption for 12 min, and then analyzed by gas chromatography-mass spectrometry (GC-MS) (Agilent 7890A and 5975C) with HP-5MS column (30 m × 0.25 mm i.d. × 0.25 μm film thickness). Oven temperature was maintained at 40 °C for 5 min and then increased to 200 °C at a rate of 5 °C/min, with the inlet temperature at 220 °C. Helium carrier gas was flowed at 1.0 mL/min with the split ratio of 1:10. The mass spectrum library (NIST 12), ion fragmentation pattern, and a reference were utilized to identify the volatile compounds separated from the total ionization chromatogram (TIC) [16]. Relative comparison was taken for the analysis of volatile compounds. The collected volatile compounds were converted into peak areas of the internal standard based on the peak areas of the sample, in order to determine the content. The retention index (RI) was determined using the Equation (2).

RI*x* = 100*n* + 100 ((*tRx* − *tRn*)/(*tRn*+1 − *tRn*)) (2)

where, RI*x* is RI of the unknown compound, *tRx* is retention time of the unknown compound, *tRn* is retention time of the *n*-alkane, and *tRn*+1 is retention time of the next *n* -alkane. *tRx* is between *tRn* and *tRn*+1 (*n* = number of carbon atoms).

GC/MS using GC-olfactometry (olfactory detection port (ODP) 3, Gerstel Co., Linthicum, MD, USA) mounted to the spectrometer was used for the sniffing test of the volatile compounds present in the coffee samples. The sniffing test was performed under the same GC/MS conditions as those of volatile compound analysis, and the test was conducted simultaneously with GC/MS analysis. Due to the solvent elution time (5 min) and possible olfactory tiredness of the panelists, the sniffing test was performed between 5 and 25 min only. Three panelists participated in the sniffing test and recorded the levels of olfactory intensity and subjectively described the characteristics of the odors. Olfactory intensities perceived by the panelists were expressed in the numbers 1 to 4 and a higher number indicates stronger olfactory intensity. [19].

Electronic tongue analysis of coffee samples was conducted using an electronic tongue module (E-tongue, ASTREE II, Alpha M.O.S, Toulouse, France) with seven sensors attached. These include two reference sensors, SPS (spiciness) and GPS (metallic taste) sensors, as well as 5 basic taste sensors, SRS (sourness), STS (saltiness), UMS (umami), SWS (sweetness) and BRS (bitterness). Each coffee sample, 50 mL, was diluted with 50 mL of distilled water and the analysis was performed in 7 repetitions. Multivariate analysis was conducted to analyze the taste patterns, and the outcomes from the sensors were expressed in relative taste scores [20].

### 2.5. Statistical Analysis

The results of this study were presented as means and standard deviations. Differences among treatments were tested using one-way analysis of variance, Tukey’s post-hoc 194 test, by SAS version 9.0 (SAS Institute Inc., Cary, NC, USA) package (*p* < 0.05).

## 3. Results and Discussion

### 3.1. Nutritional Characteristics

Compositions of constituent amino acids in the coffee samples were analyzed and the results are shown in Table 1. Glutamic acid and phenylalanine were the most abundant amino acids in all samples. Proportions of aspartic acid and glutamic acid were significantly higher in peanut coffee, (7.51 ± 0.27 in conventional peanut coffee and 8.26 ± 2.99 in high-oleic peanut coffee) and (33.33 ± 0.09 in conventional peanut coffee and 37.28 ± 1.24 in high-oleic peanut coffee), respectively. A previous study reported that aspartic acid and glutamic acid react at pH 6 in the presence of sodium to develop umami taste [21,22]. Such an increase in these amino acids in this study therefore may influence the generation of umami taste in peanut coffee. The proportion of glycine was also significantly higher in peanut coffee: 6.24 ± 0.01%, 8.66 ± 0.03%, and 7.06 ± 0.23% in normal coffee, conventional peanut coffee, and high-oleic peanut coffee, respectively. Glycine elicits sweetness [21,22], potentially leading to a difference in sweetness between control and peanut coffee. Phenylalanine, contributing to bitterness [16,22], marked the highest proportion in normal coffee (16.52 ± 0.02%) while the content decreased in peanut coffee (13.06 ± 0.17% in conventional peanut coffee and 14.67 ± 0.29% in high-oleic peanut coffee). These results are consistent with a study reporting that aspartic acid, glutamic acid, and phenylalanine contribute to typical peanut flavor [23]. These results may positively act for those who are deterred from drinking coffee due to its bitterness. Based on the result of amino acid compositions, peanut coffee would be relatively sweeter, less bitter, and more palatable (from umami taste) than normal coffee. For free amino acids, alanine was the only free amino acid found in normal coffee except for unidentified N-containing amino acids, while various amino acids were found in peanut coffee including serine, glycine, leucine, and leucine, which proves that peanut is the key source of amino acids in the coffee samples. Serine and glycine are non-essential amino acids that elicit sweetness whereas valine and leucine are essential amino acids that induce bitterness [21,22]. Valine serves as a synergistic element for umami taste [21,22]. Although phenylalanine inducing bitterness was predominant in both peanut coffees (29.60 ± 6.35% in conventional peanut coffee and 30.00 ± 4.50% in high-oleic peanut coffee), the peanut coffees showed different patterns; conventional peanut coffee had higher proportions of cysteine and hydroxylysine whereas high-oleic peanut coffee had higher proportions of valine and alanine, presumably because of differences in amino acids compositions in the two peanut cultivars [24].

Water-soluble vitamin contents in the coffee samples were determined and the results were described in Table 2. Thiamin is involved in enzyme functions, energy metabolisms from foods, appetite and digestive stimulations, and neurological functions [25]. Thiamin is known to be heat- and light-sensitive and is inactivated once the bridge of the ring structure to methylene group is cleaved, thereby making it prone to nutritional loss [16]. Peanut coffee contained significantly higher thiamin (0.09 ± 0.01 mg/100 g in conventional peanut coffee and 0.11 ± 0.00 mg/100 g in high-oleic peanut coffee) compared to normal coffee (0.06 ± 0.01 mg/100 g). These results may be attributed by the extraction method where water-soluble vitamins in peanuts were extracted through hot water extraction during the sample preparation. High-oleic peanut coffee had a significantly higher amount of thiamin compared to conventional peanut coffee (*p* < 0.05). This may be due to addition of high-oleic peanut extract that is thermally more stable than conventional peanut. A consistent result was observed by Kim et al. [16], reporting that thiamin was found in both high-oleic and conventional peanut, and roasted conventional peanut had higher decrease in thiamin than roasted high-oleic peanut, suggesting that high-oleic variety is more stable at high temperature. Riboflavin plays critical roles in maintaining energy supply and mucous membranes, producing hormones, and aiding fetal development. When deficient, it can cause oral inflammation, dermal dryness, stomatitis, and growth retardation [16,26]. Riboflavin is relatively stable to heat and oxidation but is easily destructive under alkaline conditions and ultraviolet (UV) and visible light exposure [27]. As shown in the table, only trivial amounts of riboflavin existed in all coffee samples with no statistical significance. In the study by Kim et al. [16], riboflavin content did not show significant differences between conventional and high-oleic peanuts, which is in agreement with the result of this study. Niacin controls blood circulation, redox reactions of nutrients, and liver and gland functions. Its deficiency includes digestive complications, mental impairment, and mucosal inflammation [16,28]. Niacin is thermally stable due to the heterocyclic pyrimidine ring structure and has low loss rates against external energy changes [16]. The niacin contents ranged between 0.63 and 0.82 mg/mL (*p* < 0.05), showing that peanut coffee possessed significantly higher niacin content than normal coffee (*p* < 0.05), albeit no significant difference between the peanut coffees was found (*p* > 0.05). Such higher niacin content in peanut coffee than normal coffee is attributed to niacin present in peanut, which was also confirmed by Kim et al. [16] in conventional and high-oleic peanut samples. The result highlighted that peanut added coffee would be not only a favorite beverage but also an excellent source of nutrients, particularly thiamin and niacin.

Glucose and sucrose content in the coffee samples were measured and the results were demonstrated in Table 2. Glucose content ranged from 1.46 to 2.19 mg/mL, and high-oleic peanut coffee had significantly higher glucose content than normal coffee. Sucrose content ranged from 0.07 to 1.12 mg/mL and both peanut coffees had significantly higher sucrose content than normal coffee. Such a higher sugar content is anticipated to influence the sweetness intensity of the overall coffee taste, and the innate bitterness of coffee may possibly be masked by sweetness, which can further improve the consumer preference and acceptability of coffee [29]. Hong et al. [30] found that sugar content in peanut sprout extract was elevated with heat treatment, indicating that changes in water content greatly influence sugar content. Similarly, in the present study, sugar content in conventional and high-oleic peanut presumably increased due to a decrease in water content after roasting, which may ultimately lead to the increase in sugar content in conventional and high-oleic peanut coffee.

Caffeine content in the coffee samples were analyzed and the results are shown in Table 2. The amount of caffeine contained in the samples ranged between 9.98 and 16.67 mg/mL, among which peanut coffee showed significantly lower caffeine content than normal coffee, probably because of differences in caffeine extraction rates induced by extraction solvents (water vs. peanut extract). Similarly, studies have described that a lower amount of caffeine was extracted when using non-polar solvent [31], and Gu et al. [32] observed various caffeine extraction ratios upon different extraction solvents. As caffeine is known as a bitterness stimulus, the relatively lower amount of caffeine in peanut coffee may reduce the bitterness of coffee and relieve the adverse effects of caffeine overconsumption such as disturbed sleep, anxiety, nervousness, indigestion, and caffeine addiction [33,34].

### 3.2. Physicochemical Characteristics

Antioxidant capacity was measured by DPPH radical scavenging activity and total phenolic content and the results are shown in Table 3. For DPPH radical scavenging activity, SC_50_ showed 61 ± 3.93, 55 ± 3.28, and 54 ± 0.67 mg/mL, in normal coffee, conventional peanut coffee, and high-oleic peanut coffee, respectively, indicating that antioxidant capacity is relatively higher in peanut coffee than normal coffee. Hydrogen atoms in peanuts provide reductones and decompose active oxygen chains, resulting in an increased antioxidant capacity [35]. Such a higher antioxidant capacity in peanut coffee showed a similar pattern with another study that reported an increased antioxidant capacity in *Cheonggukjang* with peanu (*Arachis hypogaea* L.) powder added [36]. Total phenolic content was shown to be highest in conventional peanut coffee, 35.78 ± 0.16 mg/mL, whereas the lowest content was in normal coffee, 29.92 ± 4.17 mg/mL. Such a higher phenolic content in the peanut coffee may be because phenolic components embedded in peanuts are extracted through hot water extraction, and this result is consistent with a study by Park [37] that analyzed total phenolic content of coffee ground residues by hot water extraction.

The pH in the present study was higher in peanut coffee (4.59 ± 0.05 for conventional peanut coffee and 4.71 ± 0.05 for high-oleic peanut coffee) compared to normal coffee (4.37 ± 0.07). pH is not influenced by extraction rate but by extraction conditions and roasting conditions [30]. Such pH difference may stem from the difference in extraction solvents used for coffee making (water vs. peanut extract), which is anticipated to have an impact on the sourness of coffee [31].

### 3.3. Sensory Characteristics

Volatile compound analysis and the sniffing test were performed by GC/MS, and the results are shown in Table 4 and Table 5. A total of 86 volatile compounds were detected in normal coffee, conventional peanut coffee, and high-oleic peanut coffee (14 alcohols, 7 aldehydes, 8 hydrocarbons, 8 acids and esters, 35 heterocyclics, and 14 ketones). Compared to normal coffee, conventional peanut coffee and high-oleic peanut coffee contained more types of alcohols, acids, esters, heterocyclics, and ketones. Maillard reaction occurs during peanut roasting and synthesizes volatile compounds, resulting in a larger number of types of volatile compounds in peanut coffee compared to normal coffee [19,30]. The present study also identified two hydrocarbons in normal coffee that were not found in peanut coffee, 6-aza-5,7,12,14-tetrathia pentadecane and β-bourbonene. These hydrocarbons may convert to other volatile compounds due to the addition of peanut extract in peanut coffee [30]. A study reported that volatile compounds react with other compounds such as amino acids, fatty acids, and sugars in food substances to develop flavors [38]. In this sense, differences in amino acids compositions, caffeine contents, and sugar contents between the peanut coffees, as shown in Table 1, may yield differences in the types of volatile compounds generated.

As shown in Table 5, some of the volatiles detected by GC were confirmed by panelists through GC-olfactometry, including 2-ethyl-3,6-dimethylpyrazine, 3-ethyl-2,5-dimethyl-pyrazine, 2,2′-methylenebis-furan and furfuryl propinoate in different odor intensities and odor descriptions. 2-Ethyl-3,6-dimethyl pyrazine was recognized at RT 17.53 by panelists using GC-Olfactometry. The odor description was like “peanut” with a rating of 1 in odor intensity. 3-Ethyl-2,5-dimethyl pyrazine was recognized at RT 17.55 by panelists. The odor description was like “sweet, peanut” with a rating of 1 in odor intensity. They all existed at a low concentrations based on the human sensory response. The retention times of 2-ethyl-3,6-dimethyl pyrazine and 3-ethyl-2,5-dimethyl pyrazine are very close, but 2-ethyl-3,6-dimethyl pyrazine was only detected in CPC, and 3-ethyl-2,5-dimethyl pyrazine was in NC and HPC. Therefore, the two close compounds did not interfere with each other, because they existed in different sample runs. Three panelists recognized two time points about “peanut” and “sweet, peanut” odors during GC-O analysis in the different sample runs. The results showed that these compounds were perceived as having sweet and peanut odors, respectively, with no differences in content. Pyrazine, a key volatile in roasted peanut and coffee odors, is generated by Maillard reaction during the roasting process and is known to have low odor thresholds [23,39]. As pyrazine rapidly increases during peanut roasting at high temperature for a long time, there may be no difference in pyrazine contents among the coffee samples [23]. The study also discovered that the amount of pyrazine formation was positively associated with the roasting time of peanut [23]. In addition to peanut, various pyrazine compounds were identified in roasted coffee bean and, among them, the pyrazine with the ethyl group showed relatively lower threshold compared to other pyrazines [12]. The literature highlighted that pyrazine complexly interacts with other volatile compounds to develop coffee flavor [7]. Recent studies also identified various pyrazines including 3-ethyl-2,5-dimethyl-pyrazine and 2-ehtyl-3,6-dimehtyl pyrazine in roasted oriental herb samples and reported roasted odor descriptions through sniffing test [19,20]. 2,2-Methylenebisfuran (17.6 min) and furfuryl propionate (17.7 min) were found in conventional peanut coffee and high-oleic peanut coffee, which perceived peanut and roasted odors in the sniffing test, respectively. Furans have low thresholds, and therefore only a small increase in furan contents allows having roast and nutty odors in conventional peanut coffee and high-oleic peanut coffee [23,40]. Higher concentrations of furans were observed in peanut coffee than normal coffee, which would attribute to the roasted odor in peanut coffee [2,7,23]. In comparison to the other volatiles found in coffee samples, caffeine existed in relatively higher concentration throughout all coffee samples (0.109 ± 0.109, 0.906 ± 0.995, and 0.127 ± 0.127 ug/100 g in normal coffee, conventional peanut coffee, and high-oleic peanut coffee, respectively), which is consistent with results of the caffeine content shown in Table 2. Caffeine is a naturally occurring compound in coffee and its content varies depending on roasting time and temperature as well as type of coffee [41]. Although caffeine was found in high concentration in this study, it is known as an odorless compound [41], and thereby was not perceivable during the sniffing test. A similar trend was also evident in a recent study showing that tridecane existed in the highest concentration, but was not detectable through sniffing test, in *Platycodon grandiflorum* roots [19].

**Figure 1:**
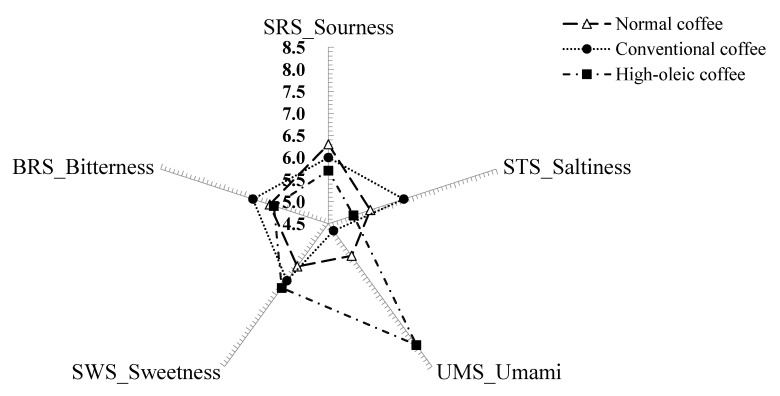
Plot of chemical sensors attributed sensory of coffee using an electronic tongue.

Electronic tongue analysis was performed to investigate the chemical sensors-assisted taste differences among the coffee and the results are shown in Figure 1. Electronic tongue analysis is a non-destructive pattern analysis method that does not require specific pretreatments and determines the sensitivity of food tastes through sensor arrays and a pattern recognition system [42]. Peanut coffee showed relatively lower sourness than normal coffee, and this is possibly associated with the increased pH in peanut coffee, as shown in Table 3. High-oleic peanut coffee exhibited lower bitterness compared to normal coffee, which may be attributed to a decrease in the phenylalanine (Table 1) and caffeine contents (Table 2) that are associated with bitterness [21]. Previous studies also demonstrated similar results, showing that changes in phenylalanine and caffeine content upon roasting were linked to the bitterness of *Platycodon grandiflorum* roots and peanut sprout extracts [19,30]. Peanut coffee showed higher saltiness, umami taste, and sweetness than normal coffee. Particularly, high-oleic peanut coffee marked the highest score in umami taste, probably because a relatively higher amount of glutamic acid in high-oleic peanut coffee (Table 3) reacts with sodium, leading to an increased umami taste [21]. Kato et al. [21] suggested that glutamic acid is representative of umami taste. Indeed, recent studies provide evidence that glutamic acid influences the umami taste sensor of electronic tongue analysis in ranges of oriental herb samples [19,20,22]. Peanut coffee exhibited higher sweetness than normal coffee, which coincides with the result of increased glucose, sucrose, and glycine contents in peanut coffee (Table 2). This result is consistent with a recent study showing that roasted peanut sprout extracts showed higher brix than the non-roasted extracts [30]. In regard to amino acids associated with sweetness, Lee et al. [19] reported that sweetness increased with an increased level of glycine in electronic tongue analysis of roasted *Platycodon* sample. The taste result by electronic tongue not only comes from major taste components of peanut but is an expression of a complex interaction of tastes from coffee and peanuts [43]. Electronic tongue analysis can exclude the subjectivity of panelists and offers analysis results within a short time and has therefore been widely utilized in recent years [43]. However, electronic tongues lack the capacity to reflect all the complexity of biological aspects associated with the human oral environment. Therefore, future studies are warranted in investigating links between the sensory aspects of foods obtained through electronic tongue and human tongue from panelists.

## 4. Conclusions

In order to broaden the utilization of peanut that possesses potential nutritional and health benefits, this study prepared coffee with conventional peanut and high-oleic peanut extracts by hot water extraction and investigated nutritional and physicochemical parameters, volatile compounds, taste, and flavor. Overall, peanut coffee was superior in type and content of amino acids, thiamin and niacin content, glucose and sucrose content, and antioxidant capacity in comparison to normal coffee. Furthermore, peanut coffee showed an increased sweetness and umami taste and possessed volatile compounds that induced roasted and peanut odors. To our knowledge, this is the first study that prepared coffee with peanut extracted water and determined various parameters from a nutrition and food chemistry perspective. Future studies need to optimize the extraction conditions to generate desirable sensory characteristics and confirm detailed mechanisms of volatile compound generation in developed coffee. More studies are also warranted, in the investigation of sensory attributes of the developed coffee, with sensory panels. This study will be useful for researchers who are interested in creating coffee flavors, understanding the nutritional quality of peanuts from breeding improvement, and broadening peanut utilization other than snacks.

## Figures and Tables

**Table 1:** Amino acid compositions in coffee samples.

**Amino Acid**	**Constituent Amino Acid Composition(%)**		
	**Normal Coffee**	**Conventional Peanut Coffee**	**High-Oleic Peanut Coffee**
Aspartic acid	5.42 ± 0.06 ^a (1)^	7.51 ± 0.27 ^a^	8.26 ± 2.99 ^a^
Threonine	N.D. ^(^^2^^)^	0.80 ± 0.12 ^a^	N.D.
Serine	0.80 ± 0.01 ^c^	2.65 ± 0.06 ^a^	1.37 ± 0.10 ^b^
Glutamic acid	32.82 ± 0.19 ^b^	33.33 ± 0.09 ^b^	37.28 ± 1.24 ^a^
Glycine	6.24 ± 0.01 ^c^	8.66 ± 0.03 ^a^	7.06 ± 0.23 ^b^
Alanine	3.74 ± 0.02 ^b^	4.03 ± 0.01 ^a^	3.52 ± 0.18 ^b^
Cysteine	13.63 ± 0.07 ^a^	9.15 ± 0.21 ^c^	10.59 ± 0.32 ^b^
Valine	2.56 ± 0.03 ^a^	2.45 ± 0.00 ^a^	1.79 ± 0.10 ^b^
Leucine	N.D.	1.96 ± 0.00 ^a^	N.D.
Tyrosine	11.81 ± 0.31 ^a^	8.42 ± 0.23 ^c^	9.53 ± 0.08 ^b^
Phenylalanine	16.52 ± 0.02 ^a^	13.06 ± 0.17 ^c^	14.67 ± 0.29 ^b^
Arginine	N.D.	2.14 ± 0.02 ^a^	N.D.
Hydroxylysine	N.D.	0.63 ± 0.03 ^a^	0.37 ± 0.26 ^ab^
Unidentified amino acids	6.45 ± 0.02 ^a^	5.19 ± 0.00 ^c^	5.67 ± 0.24 ^b^
Total	100	100	100
**Amino Acid**	**Free Amino Acid Composition(%)**		
	**Normal Coffee**	**Conventional Peanut Coffee**	**High-Oleic Peanut Coffee**
Serine	N.D.	2.80 ± 0.66 ^a^	2.10 ± 0.41 ^b^
Glycine	N.D.	3.95 ± 0.74 ^a^	4.19 ± 1.46 ^a^
Alanine	5.10 ± 0.06 ^b^	5.69 ± 1.16 ^b^	10.13 ± 1.51 ^a^
Valine	N.D.	10.29 ± 1.97 ^a^	14.98 ± 4.59 ^a^
Cysteine	N.D.	15.93 ± 3.45 ^a^	8.03 ± 5.68 ^ab^
Isoleucine	N.D.	1.21 ± 0.55 ^a^	N.D.
Leucine	N.D.	3.66 ± 0.68 ^b^	4.51 ± 0.67 ^a^
Phenylalanine	N.D.	29.60 ± 6.35 ^a^	30.00 ± 4.50 ^a^
Hydroxylysine	N.D.	12.84 ± 8.16 ^a^	N.D.
Unidentified amino acids	94.90 ± 0.25 ^a^	14.01 ± 2.60 ^c^	28.42 ± 4.07 ^b^
Total	100	100	100

Data are given as mean ± SD values from experiments performed in duplicate. ^(1)^ Mean values with different letters within the same row are significantly different according to Tukey’s post-hoc 194 test (*p* < 0.05).^(2)^ N.D.: not detected.

**Table 2:** Nutritional constituents in coffee samples.

Sample	Thiamine	Riboflavin	Niacin	Glucose	Sucrose	Caffeine
	(mg/100 g)	(mg/100 g)	(mg/100 g)	(mg/mL)	(mg/mL)	(mg/mL)
Normal coffee	0.06 ± 0.01 ^c (1)^	N.D. ^(2)^	0.63 ± 0.05 ^b^	1.46 ± 0.08 ^b^	0.07 ± 0.07 ^c^	16.67 ± 0.38 ^a^
Conventional peanut coffee	0.09 ± 0.01 ^b^	N.D.	0.82 ± 0.01 ^a^	1.67 ± 0.11 ^b^	1.12 ± 0.03 ^a^	15.00 ± 0.08 ^b^
High-oleic peanut coffee	0.11 ± 0.00 ^a^	N.D.	0.78 ± 0.02 ^a^	2.19 ± 0.16 ^a^	0.68 ± 0.15 ^b^	9.98 ± 0.27 ^c^

Data are given as mean ± SD values from experiments performed in triplicate. ^(1)^ Mean values with different letters within the same row are significantly different according to Tukey’s post-hoc 194 test (*p* < 0.05).^(2)^ N.D.: not detected.

**Table 3:** Physiochemical characteristics in the three kinds of coffee.

Sample	DPPH-SC_50_^(1)^	TPC ^(2)^	pH
	(mg)	(mg/mL)	
Normal coffee	61 ± 3.93 ^a (3)^	29.92 ± 4.17 ^a^	6.37 ± 0.07 ^b^
Conventional peanut coffee	55 ± 3.28 ^a^	35.78 ± 0.16 ^a^	6.59 ± 0.05 ^a^
High-oleic peanut coffee	54 ± 0.67 ^a^	34.31 ± 0.67 ^a^	6.71 ± 0.04 ^a^

Data are given as mean ± SD values from experiments performed in triplicate.^(1)^ The half scavenging activity concentration by DPPH assay. ^(2)^ TPC: total phenolic content. ^(3)^ Mean values with different letters within the same row are significantly different according to Tukey’s multiple range test (*p* < 0.05).

**Table 4:** Volatile compounds in the normal-, conventional-, and high-oleic coffee samples.

Volatile Compounds	RT ^(1)^	RI ^(2)^	NC ^(3)^	CPC ^(4)^	HPC ^(5)^	I.D. ^(6)^
	(min)		(μg/100 g)	(μg/100 g)	(μg/100 g)	
Alcohols(14)						
2-Furanmethanol	10.25	878	N.D. ^(^^7^^)^	0.009 ± 0.007	N.D.	MS ^(^^8^^)^
3-Pyridinol	18.11	1119	N.D.	0.005 ± 0.007	N.D.	MS
Maltol	18.54	1134	N.D.	0.004 ± 0.006	N.D.	MS/RI
4-Amino-2,6-dimethylphenol	20.78	1210	N.D.	N.D.	0.004 ± 0.006	MS
1,2-Benzenediol	20.90	1215	N.D.	0.003 ± 0.004	N.D.	MS
5-Endo-hydroxy-protoadamantane	20.92	1215	N.D.	N.D.	0.002 ± 0.002	MS
4-Vinylphenol	21.45	1235	N.D.	N.D.	0.007 ± 0.004	MS
2-Naphthalenol	21.67	1243	N.D.	N.D.	0.002 ± 0.002	MS
4-Ethyl-2-methoxyphenol	23.20	1297	N.D.	N.D.	0.006 ± 0.002	MS
2-Methoxy-benzeneethanol	23.21	1298	N.D.	0.002 ± 0.003	N.D.	MS
4-Ethyl-2-methoxy-phenol	23.22	1298	0.002 ± 0.003	N.D.	N.D.	MS
2-Methoxy-4-vinylphenol	24.14	1335	0.014 ± 0.006	0.018 ± 0.001	N.D.	MS
2-Allyl-3-methoxyphenol	25.46	1385	0.002 ± 0.002	N.D.	N.D.	MS
Eugenol	25.46	1385	N.D.	N.D.	0.005 ± 0.003	MS/RI
Aldehydes(7)						
5-Methyl-2-furancarboxaldehyde	13.85	984	0.001 ± 0.001	N.D.	N.D.	MS
5-Methyl-2-furfural	13.90	986	N.D.	0.003 ± 0.002	0.003 ± 0.004	MS
5-(Dimethylamino)pent-2-en-4-ynal	17.37	1094	0.003 ± 0.005	N.D.	N.D.	MS
Nonanal	18.24	1124	0.005 ± 0.007	N.D.	N.D.	MS/RI
3-Hexene-1,6-dialdehyde	25.31	1379	0.014 ± 0.019	N.D.	N.D.	MS
4-Methyl-2,5-dimethoxybenzaldehyde	30.23	1581	N.D.	0.002 ± 0.002	N.D.	MS
5-Methylisophthalaldehyde	30.25	1582	N.D.	0.001 ± 0.001	N.D.	MS
Hydrocarbons(8)						
1,2-Bis(trimethylsilyl)benzene	9.36	853	N.D.	N.D.	0.001 ± 0.001	MS
Toluene-2,4-diamine	15.19	1026	N.D.	N.D.	0.003 ± 0.004	MS
2-Menthene	18.19	1122	N.D.	0.002 ± 0.003	N.D.	MS
6-Aza-5,7,12,14-tetrathia pentadecene	19.81	1177	0.008 ± 0.011	N.D.	N.D.	MS
1-(1-Propynyl)-cyclohexene	21.45	1235	N.D.	0.002 ± 0.003	N.D.	MS
β-Bourbonene	23.11	1294	0.002 ± 0.003	N.D.	N.D.	MS
2-Methyladamantane	23.89	1325	N.D.	N.D.	0.003 ± 0.004	MS
3,4-Dimethoxystyrene	25.46	1385	N.D.	0.002 ± 0.002	N.D.	MS
Acids and esters(8)						
Cascarillic acid	18.26	1125	N.D.	N.D.	0.002 ± 0.003	MS
Methyl N-hydroxybenzenecarboximidoate	12.09	933	N.D.	0.020 ± 0.029	N.D.	MS
Quinic acid	30.94	1612	N.D.	0.031 ± 0.044	N.D.	MS
Isobutyric Acid	30.96	1612	N.D.	N.D.	0.004 ± 0.006	MS
1-O-Ethyl 2-O-[2-(2-nitrophenyl)ethyl] benzene-1,2-dicarboxylate	30.90	1609	N.D.	N.D.	0.002 ± 0.002	MS
Diethyl 2-hydroxy-3-(tetrahydrofuran-2-yl)succinate	30.98	1613	N.D.	N.D.	0.005 ± 0.008	MS
2,2,4-Trimethyl-pentan-1,3-diol diisobutyrate	30.98	>1700	0.003 ± 0.005	N.D.	N.D.	MS
Phthalic acid, 5-methylhex-2-yl butyl ester	38.89	>1700	N.D.	N.D.	0.002 ± 0.002	MS/RI
Heterocyclics(35)						
N-ethyl-1,3-dithioisoindoline	8.92	840	0.007 ± 0.010	N.D.	N.D.	MS
2-Methyl-1-vinylimidazole	12.19	936	N.D.	N.D.	0.000 ± 0.001	MS
Furfuryl acetate	14.76	1099	0.001 ± 0.001	0.002 ± 0.003	N.D.	MS
3-Butynyl 4-(N-methylacetamido) benzenesulfonate	15.16	1025	0.001 ± 0.001	N.D.	N.D.	MS
Ethyl 2-(3,4-dimethylanilino)-2-oxoacetate	16.58	1430	0.075 ± 0.106	N.D.	N.D.	MS
2-Ethyl-3,6-dimethylpyrazine	17.53	1098	N.D.	0.007 ± 0.002	N.D.	MS
3-Ethyl-2,5-dimethyl-pyrazine	17.545	1199	0.007 ± 0.007	N.D.	0.007 ± 0.001	MS
Di-α-furylmethane	17.63	1102	N.D.	0.001 ± 0.002	N.D.	MS
2,2′-Methylenebis-furan	17.66	1103	0.002 ± 0.003	0.005 ± 0.007	0.014 ± 0.003	MS
Furfuryl propinoate	17.75	1106	N.D.	0.001 ± 0.002	0.003 ± 0.005	MS
4-Ethyl-2,5,6-trimethylpyrimidine	19.84	1178	N.D.	0.005 ± 0.001	N.D.	MS
5-Methyl-2-furfurylfuran	20.48	1199	N.D.	N.D.	0.010 ± 0.001	MS
2-(2-furanylmethyl)-5-Methylfuran	20.50	1202	0.010 ± 0.004	0.004 ± 0.006	N.D.	MS
N-Furfuryl pyrrole	20.54	1201	N.D.	0.003 ± 0.003	0.016 ± 0.004	MS
1-(2-furanylmethyl)-1H-Pyrrole	20.57	1211	0.014 ± 0.008	0.008 ± 0.011	N.D.	MS
3-Ethyl-2-formylthiophene	20.80	1211	N.D.	0.003 ± 0.004	N.D.	MS
2,3-Dihydro-6-methylthieno [2,3c]furan	20.80	1211	0.002 ± 0.003	N.D.	N.D.	MS
2-Dimethylamino-4,5-dimethyl-oxazole	20.81	1211	N.D.	N.D.	0.003 ± 0.005	MS
2,3-Dihydrobenzofuran	21.45	1235	N.D.	0.006 ± 0.009	N.D.	MS
Furfuryl isovalerate	21.58	1239	N.D.	N.D.	0.002 ± 0.003	MS
Furfuryl 3-methyl butanoate	21.58	1240	0.002 ± 0.002	0.001 ± 0.002	0.002 ± 0.002	MS
2-(2′,4′,5′-trimethylphenyl)-Propylene oxide	23.11	1294	N.D.	0.002 ± 0.004	N.D.	MS
2,2′-(oxydimethylene)Di furan	23.78	1320	N.D.	0.009 ± 0.003	0.013 ± 0.003	MS
2,2′-Difurfuryl ester	23.80	1436	0.006 ± 0.003	N.D.	N.D.	MS
2-Methyl-6-hydroxyquinoline	24.98	1367	N.D.	N.D.	0.003 ± 0.004	MS
5-Methyl[1,2,4]triazolo[1,5-a]pyrimidine	26.21	1414	N.D.	N.D.	0.001 ± 0.001	MS
1-Furfuryl-2-formyl pyrrole	26.72	1495	0.008 ± 0.003	0.008 ± 0.002	0.012 ± 0.006	MS
Pyroquilon	27.27	1458	N.D.	N.D.	0.003 ± 0.005	MS
3,4,5,6-Tetrahydro-2H-1,6-benzoxazocine	27.49	1462	N.D.	N.D.	0.005 ± 0.001	MS
1-Furfuryl-2-acetyl pyrrole	28.20	1611	0.002 ± 0.003	0.003 ± 0.004	0.010 ± 0.008	MS
Ethyl phthalate	30.92	1613	0.002 ± 0.000	0.002 ± 0.003	N.D.	MS
Caffeine	36.26	>1700	0.109 ± 0.020	0.067 ± 0.001	0.089 ± 0.055	MS
Butyl phthalate	38.88	>1700	0.004 ± 0.006	N.D.	N.D.	MS
Dibutyl phthalate	38.88	>1700	N.D.	0.003 ± 0.005	N.D.	
Phthalic acid, 2-ethylhexyl isohexyl ester	38.88	>1700	0.001 ± 0.002	N.D.	N.D.	MS
Ketones(14)						
Butyrolactone	12.52	946	N.D.	0.002 ± 0.003	N.D.	MS
3-Chromanone	17.69	1104	0.002 ± 0.002	N.D.	N.D.	MS
5,5,6-Trimethylbicyclo[2.2.1]heptan-2-one	22.41	1270	N.D.	N.D.	0.002 ± 0.003	MS
3′,5′-Dihydroxyacetophenone	23.15	1296	N.D.	0.002 ± 0.002	N.D.	MS
7,7-Dimethylbicyclo[3.3.0]octan-2-one	23.19	1297	N.D.	0.001 ± 0.002	N.D.	MS
4′-Methoxyacetophenone	23.90	1325	N.D.	N.D.	0.001 ± 0.002	MS
1-(5(methyl-2-furanyl)-1-Buten-3-one	23.90	1325	N.D.	0.002 ± 0.002	N.D.	MS
2(1H)-Leoidinone	24.99	1368	N.D.	0.001 ± 0.002	N.D.	MS
2-Methyl-4-quinazolinone	25.19	1375	N.D.	N.D.	0.002 ± 0.002	MS
4-Methyl-6(2-methylpropenyl)-2H-pyran-2-one	25.45	1385	0.002 ± 0.002	N.D.	N.D.	MS
*β*-Damascenone	26.00	1406	0.003 ± 0.005	0.003 ± 0.004	0.003 ± 0.004	MS/RI
Megastigmatrienone	30.64	1598	0.002 ± 0.003	0.005 ± 0.001	0.006 ± 0.008	MS
3,6,7,8-Tetrahydro-5-propyl-2H-azulen-1(2H)-one	31.68	1644	N.D.	0.003 ± 0.004	N.D.	MS
2-Methylenecyclododecanone	37.83	>1700	N.D.	0.003 ± 0.004	N.D.	MS

Data values are given as mean ± SD from experiments performed in duplicate. ^(1)^ RT: retention time, ^(2)^ RI: retention index, ^(3)^ NC: normal coffee, ^(4)^ CPC: conventional peanut coffee, ^(5)^ HPC: High oleic peanut coffee, ^(6)^ ID: identification, ^(^^7)^ N.D.: not detected. ^(^^8)^ MS, RI: mass spectrometry, retention index.

**Table 5:** Sensory description and odor intensity of the normal-, conventional-, and high-oleic coffee samples using gas chromatography (GC)-olfactometry.

Major Compounds	RT ^(1)^	RI ^(2)^	Odor Intensity	Odor Description			I.D. ^(6)^
	(min)		NC ^(3)^	CPC ^(4)^	HPC ^(5)^		
2-Ethyl-3,6-dimethy pyrazine	17.53	1098	N.D. ^(^^7^^)^	1	N.D.	Peanut	MS/GC-O ^(^^8^^)^
3-Ethyl-2,5-dimethyl pyrazine	17.55	1199	1	N.D.	1	Sweet, Peanut	MS/GC-O
2,2′-Methylenebis-furan	17.66	1103	1	1	1	Roasted, Peanut	MS/GC-O
Furfuryl propinonate	17.75	1106	N.D.	1	1	Roasted	MS.GC-O

Data values are given as mean ± SD from experiments performed in duplicate. ^(1)^ RT: retention time, ^(2)^ RI: retention index, ^(3)^ NC: normal coffee, ^(4)^ CPC: conventional peanut coffee, ^(5)^ HPC: high oleic peanut coffee, ^(6)^ ID: identification, ^(^^7^^)^ N.D.: not detected. ^(^^8)^ MS, GC-O: mass spectrometry, gas chromatography-olfactometry.
